# A Fluorescent Assay to Search for Inhibitors of HIV-1 Integrase Interactions with Human Ku70 Protein, and Its Application for Characterization of Oligonucleotide Inhibitors

**DOI:** 10.3390/biom10091236

**Published:** 2020-08-25

**Authors:** Simon Galkin, Anna Rozina, Arthur Zalevsky, Marina Gottikh, Andrey Anisenko

**Affiliations:** 1 Faculty of Bioengineering and Bioinformatics, Lomonosov Moscow State University, 119992 Moscow, Russia; simon.galkin@gmail.com (S.G.); anarou@inbox.ru (A.R.); aozalevsky@gmail.com (A.Z.); 2 Shemyakin and Ovchinnikov Institute of Bioorganic Chemistry of the Russian Academy of Sciences, 117997 Moscow, Russia; 3 Chemistry Department, Lomonosov Moscow State University, 119992 Moscow, Russia; gottikh@belozersky.msu.ru; 4 Belozersky Institute of Physico-Chemical Biology, Lomonosov Moscow State University, 119992 Moscow, Russia

**Keywords:** HIV-1 integrase, Ku70, oligonucleotide inhibitors, protein-protein interaction inhibitors, fluorescence assay, fluorescent tags, high-throughput screening

## Abstract

The search for compounds that can inhibit the interaction of certain viral proteins with their cellular partners is a promising trend in the development of antiviral drugs. We have previously shown that binding of HIV-1 integrase with human Ku70 protein is essential for viral replication. Here, we present a novel, cheap, and fast assay to search for inhibitors of these proteins’ binding based on the usage of genetically encoded fluorescent tags linked to both integrase and Ku70. Using this approach, we have elucidated structure-activity relationships for a set of oligonucleotide conjugates with eosin and shown that their inhibitory activity is primarily achieved through interactions between the conjugate nucleic bases and integrase. Molecular modeling of HIV-1 integrase in complex with the conjugates suggests that they can shield E212/L213 residues in integrase, which are crucial for its efficient binding to Ku70, in a length-dependent manner. Using the developed system, we have found the 11-mer phosphorothioate bearing 3’-end eosin-Y to be the most efficient inhibitor among the tested conjugates.

## 1. Introduction

Nowadays human immunodeficiency virus type 1 (HIV-1) reproduction can be controlled by specific antiretroviral therapy (ART). As a result, HIV-induced infection is reclassified to a manageable chronic disease [1]. However, ART usage is associated with the risk of the development of resistant viral strains due to the low fidelity of HIV-1 reverse transcriptase [2,3,4]. Unfortunately, these strains are increasingly common in ART-naïve patients [5], making the development of new approaches to block HIV-1 replication an urgent issue. In the present study, we describe a system that enables the screening of potential inhibitors of protein–protein interaction and tested it on modified oligonucleotides.

HIV-1 requires many cellular factors in order to successfully complete its replication [6,7,8]. Identification of these host cell factors and elucidation of their role in HIV-1 replication can reveal new targets for anti-HIV therapeutics that will overcome the viral resistance to existing drugs [8]. Some of the known cellular factors are involved in HIV-1 replication due to their direct interactions with viral proteins, e.g., HIV-1 integrase (IN) [6]. One of the well-studied IN partners is cellular protein LEDGF/p75, which both enhances integration efficiency and preferentially guides HIV-1 integration to actively transcribed genes [9,10,11,12]. To interfere with this interaction, a family of allosteric inhibitors called LEDGINs (after the lens epithelium-derived growth factor/p75 cofactor binding pocket on IN) was recently developed and characterized [12,13]. Since the LEDGINs binding pocket at the IN dimer interface is distant from the site of canonical inhibitors binding (e.g., raltegravir and dolutegravir), these compounds have overcome resistance to classical IN inhibitors [14]. Moreover, the use of such inhibitors is expected to cause a minimal chance of resistance development due to the high conservativeness of amino acid residues on the surface of two proteins interaction. 

Another cellular protein Ku70 has been also identified as a host partner for HIV-1 IN [15,16,17]. It protects IN from proteasomal degradation by an unknown mechanism [15] and participates in post-integrational DNA repair [17]. In human cells Ku70 forms a heterodimeric complex with the Ku80 subunit. As a component of DNA-dependent protein kinase (DNA-PK), the Ku heterodimer plays a key role in the repair of double-strand DNA breaks (DSB) by the non-homologous end joining (NHEJ) pathway and in V(D)J and class switch recombination [18,19]. Despite the absence of DSB in the HIV-1 integration intermediate (a product of viral DNA insertion into the cell genome produced by IN), the interaction of IN and Ku70 promotes recruitment of the functional DNA-PK complex, the phosphorylation activity of which is necessary for post-integrational DNA repair [17]. Recently, we have identified point amino acid mutations in IN, which prevent Ku70 binding [16] and impair HIV-1 replication by affecting post-integrational DNA repair [17]. Thereby, the search for inhibitors impairing the interaction between IN and Ku70 is a relevant task.

Although structures of Ku70/Ku80 heterodimer alone (e.g., PDB ID: 1JEQ, 1JEY) or in macromolecular complexes (e.g., PDB ID: 5Y3R, 6ERF-H) are available, neither the full-size structure of native HIV integrase nor its complex with Ku70 is determined yet due to low solubility of HIV-1 IN. As the structure of these proteins complex is yet unknown, the search for inhibitors of their interaction can only be performed by screenings. To date, there is a broad spectrum of high-throughput methods of search for inhibitors of protein-protein interactions (PPI), such as AlphaScreen, precipitation with subsequent ELISA or bioluminescence detection of prey protein, etc. [20,21,22,23,24,25]. However, they involve additional reagents, such as antibodies and special resins, which increases the cost of the screening procedure. Another approach called fluorescence-based protein-protein interaction assay (FluorIA) implies testing the interaction between two proteins, one of which is immobilized on a plate, and the other contains genetically encoded fluorescent tag, e.g., enhanced green fluorescent protein (eGFP) [26]. Using fluorescent proteins (FP) as tags significantly simplifies the analysis and reduces its cost.

We have modified the FluorIA concept and designed a method of search for the inhibitors of the interaction between IN and Ku70. In particular, we used FP-tags attached to both IN and Ku70, which allows additional control of the bait protein levels. Normalization of the prey protein fluorescence level by the bait protein fluorescence level increases accuracy of the quantitative analysis of the characteristics of the inhibitor, for instance, IC50. We have shown that this approach is also applicable to the analysis of the effects of fluorescent inhibitors. In this case, the protein interaction is analyzed by glutathione sepharose affinity chromatography with subsequent sample separation in SDS-PAGE, which decreases the performance, but allows distinguishing inhibitor fluorescence from that of the proteins of interest.

Using our method, we managed to study the structural peculiarities of the activity of the previously described inhibitors of IN and Ku70 interaction–conjugates of oligonucleotides with eosin-Y. We showed before that the conjugate of the 11-mer 2′-O-methyl-oligonucleotide GGUUUUUGUGU with eosin-Y (11-OM-E) hinders the interaction between IN and Ku70, and the disruption of the interaction requires both the oligonucleotide moiety of the inhibitor and the eosin-Y [16]. The interaction of the inhibitor with the IN has been closely studied by modeling the structures of IN complexes with 11-OM-E derivates. The Ku70 binding site in IN has been verified to be shielded by these inhibitors. Therefore, our novel data improve the accuracy of the model of 11-OM-E interactions with IN enabling targeted inhibitor design via in silico search of compounds with high affinity to the same site in IN in the future.

## 2. Materials and Methods

Oligonucleotides and plasmids: All oligonucleotides were synthesized by the phosphoramidite method. Modified oligonucleotides were synthesized as described earlier [27,28]. 

pET-15b_His_6_-Ku70 coding His_6_-Ku70 was produced by cloning the Ku70 gene into pET-15b to multiple cloning site. Vector pET-15b_His_6_-Ku70-tRFP coding His_6_-Ku70-tRFP was produced by cloning tRFP gene in pET15b_His_6_-Ku70 after Ku70 gene. For cloning, two PCR primers containing the BamHI restriction site C-fus-RFP_left 5′-ATGATCGGATCCAGCGGCGGCGAGCTGATCAAG-3′ and C-fus-RFP_right 5′-ATGATCGGATCCTCATCTGTGCCCCAGTTTGCT-3′ were designed to amplify the tRFP coding sequence. pET15b_His_6_-Ku70 was digested with BamHI and ligated to the digested PCR product by T4 DNA ligase (Thermo Scientific). 

Plasmid pGGWA-GST-IN was a kind gift of Dr. Marc Ruff (IGBMC, Strasbourg, France). Plasmid construct pGGWA_GST-mCer-IN coding GST-mCer-IN was produced by cloning the mCer gene into the pGGWA_GST-IN plasmid between GST and IN sequences. For cloning, two PCR primers containing the KpnI site N_CFP_Kpn1 5′-ATGATCGGTACCGGATCCGTGAGCAAGGGCGAGGAG-3′ and the NdeI site N_CFP_Nde1 5′-ATGATCCATATGTCCAGATCCCTTGTACAGCTCGTCCATGCC-3′ restriction sites were designed to amplify the mCer coding sequence. pGGWA_GST-IN was digested with NdeI/KpnI and ligated to the digested PCR product by T4 DNA ligase (Thermo Scientific, Vilnius, Lithuania).

Construct pGGWA_GST-mCer was prepared from the pGGWA_GST-mCer-IN plasmid by the addition of a STOP codon after the mCer gene by site-directed mutagenesis with primers mCer_stop_a 5′-CATGGACGAGCTGTACAAGTGATCTGGACATATGTTTTT-3′ and mCer_stop_as 5′-AAAAACATATGTCCAGATCACTTGTACAGCTCGTCCATG-3′.

Recombinant proteins expression and purification: GST-mCer-IN and His_6_-Ku70-tRFP were expressed and purified in the same way as His_6_-IN and GST-Ku70, respectively, as previously described in [29,30]. GST-mCer was produced and purified in the same way as GST-mCer-IN. 

Protein binding assay: To detect interactions between GST-mCer-IN and His_6_-Ku70-tRFP the GST-pull-down assay was performed. GST-mCer-IN and His_6_-Ku70-tRFP were incubated in 150 μL of buffer A (20 mM Hepes pH 7.5, 100 mM NaCl, 7.5 mM MgCl_2_, 2 mM 2-mercaptoethanol, 50 μg/mL BSA) at room temperature for 1 h. Then 20 μL of glutathione-agarose was added to the reaction mixtures followed by 1-h incubation at room temperature under rotation. Beads were washed twice with washing buffer (buffer A without BSA). The proteins were eluted from the beads with 20 μL of 1X SDS-PAGE loading buffer for 5 minutes and analyzed by SDS-PAGE with subsequent Western blotting or Fluorescence gel imaging. To investigate the influence of oligonucleotide inhibitors on the His_6_-Ku70-tRFP/GST-mCer-IN complex stability, indicated oligonucleotides were incubated in increasing concentrations (0–3200 nM) with 200 nM GST-mCer-IN and 200 nM His_6_-Ku70-tRFP in 150 µL of buffer A at room temperature for 1 h. Then the complexes His_6_-Ku70-tRFP/GST-mCer-IN and free GST-mCer-IN were precipitated by glutathione-agarose as described above. After elution of proteins with 20 μL of 1× SDS-PAGE the levels of His_6_-Ku70-tRFP and GST-mCer-IN were analyzed by standard SDS-PAGE electrophoresis with subsequent detection of fluorescence in the gel (see Fluorescence imaging subsection). The fluorescence signals ratios (tRFP/mCer) were used as a measure of proteins binding efficiency. This ratio in the absence of the inhibitor was taken as 100%.

To detect interactions between GST-mCer-IN and His_6_-Ku70-tRFP using Well-Coated™ Glutathione plates (G-BIOSCIENCES), GST-mCer-IN and His_6_-Ku70-tRFP were incubated in plate wells in 150 μL of buffer A at room temperature for 2 h under rotation. Then wells were washed twice with washing buffer (buffer A without BSA) and fluorescence was measured.

Western blot analysis: Protein samples were separated by 12% SDS PAGE and analyzed for the presence of GST- or His_6_-tag by WB with rabbit anti-GST (Sigma) and mouse anti-His_6_ antibodies (Sigma), respectively.

Fluorescence imaging: Fluorescent signals were measured in gel using the ChemiDoc MP system (Bio-Rad) or in black 96-well plates using VICTOR Multilabel Plate Reader (PerkinElmer). In the case of gel fluorescence measuring, 530/28 and 605/50 nm emission filters and Blue Epi illumination and Green Epi illumination excitation sources were used for mCer and tRFP, respectively. In the case of plates fluorescence measuring, P430/F460 and F555/610 excitation/emission filters were used for mCer and tRFP, respectively.

Molecular dynamics: Initial DNA oligonucleotides in A and B forms were generated with Web-3DNA v 2.0 [31]. Then the fusion with complex of HIV-1 integrase (PDB ID: 1EX4, residues 206–270) and eosin-Y+linker was made with PyMOL Molecular Graphics System, Version 2.0.7 Schrödinger, LLC (PyMOL). Complex of HIV-1 integrase and eosin-Y+linker has been obtained by a flexible docking earlier [16]. ACE cap was added to the protein N-terminus via PyMOL. To increase the sampling efficiency, a set of starting structures was generated by rotating an oligonucleotide around a specific bond (O5′-P of the first nucleotide for both forms in the distal site and B-form in the proximal site; P of the first and O3’ of the second nucleotide for A-form in the proximal site) with a step of 20 degrees resulting in 18 rotamers for each system. The difference in the choice of bonds is due to the geometry features of the A and B-forms. The bonds were chosen so that the starting structures would cover the maximum area. This procedure was performed with PyMol. All molecular dynamics simulations were done with GROMACS v 5.1.2 [32] and amber14sb_OL15 force field, in 100 replicas for each starting structure (Supplementary Figure S1). The simulated-annealing-like procedure included 100 ps molecular dynamics simulation with 1 fs step size in a vacuum and electrostatic interactions as main acting forces. Initial velocities were independently generated for every run. Protein and oligonucleotide were divided into two temperature coupling groups with reference temperatures of 10 K and 400 K respectively with the Velocity-rescale thermostat. An oligonucleotide was heated to 1000 K in 5 ps, then cooled to 100 K in the next 45 ps following 50 ps equilibration. Group cut-off scheme was employed. After the simulations, minimal distances between oligonucleotides and the amino acids were calculated with python tools ProDY [33] and pyRMSD [34]. Graphs were plotted with GraphPad Prism v 7.03 and python modules Pandas v 0.24.2 [35], Seaborn v 0.9.0, and Matplotlib v 3.0.3 [36]. Visualization of biomolecules was performed with the PyMOL.

## 3. Results

### 3.1. Design and Validation of a System for Search for Inhibitors of the Interaction between HIV-1 Integrase and Ku70

As noted above, to date, there is a rather wide range of methods of PPI analysis and of the search for their inhibitors. These include such classical approaches as pull-down with a subsequent Western blot or ELISA test, as well as more advanced systems, such as Alpha Screen [20,21,22,23,24,25]. Unfortunately, they require additional reagents to detect protein interactions. In the former case, these are antibodies, and in the latter, these are donor and acceptor beads, which significantly increases the cost of screening.

To search for inhibitors of the interaction between IN and Ku70, we have designed a system based on the already published FluorIA system [26], which is based on using a fluorescent protein tag attached to one of the interacting proteins. We have modified this system and suggest using two different fluorescent genetically encoded tags on the prey and bait proteins, in our case Ku70 and IN, respectively. One of the tags is needed to estimate the levels of complex formation, and the other is necessary for the normalization by the bait protein level. The important limitation of fluorescent tags usage is the potential interfering of the tags with proper proteins binding due to the sterical hindrance of binding sites. The possibility of such tags usage should be tested additionally in a particular case. Another limitation is the need to use mild conditions for elution of proteins from affine sorbents since the FP fluorescence is sensitive to heating, significant changes in pH, etc. [37].

**Figure 1:**
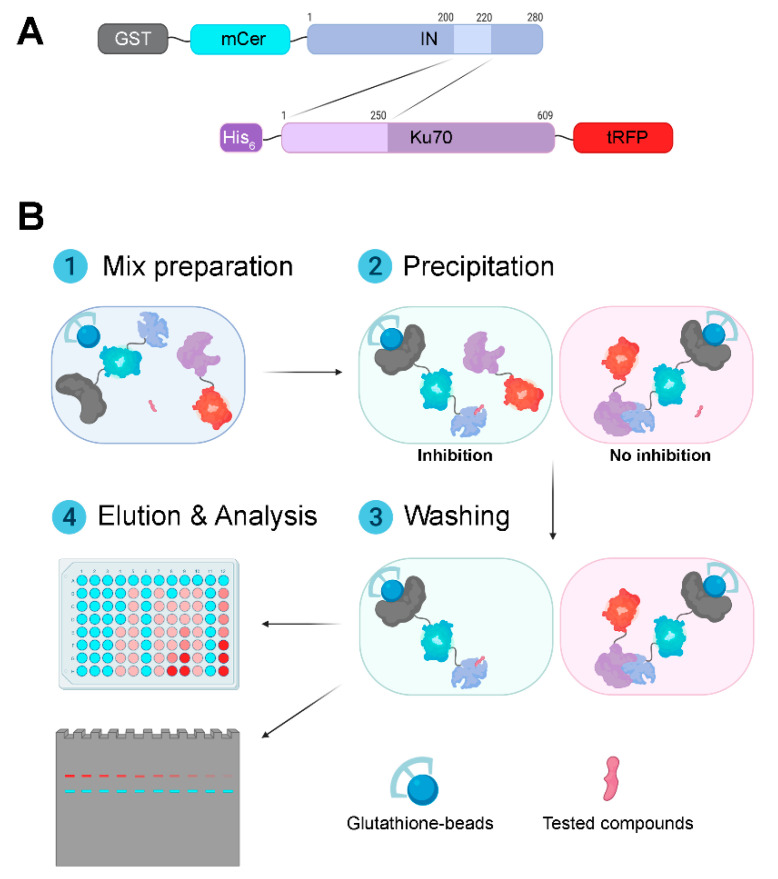
General scheme of the fluorescent assay for the discovery of Ku70-IN inhibitors. (**A**) Schematic illustration of recombinant Ku70 and HIV-1 integrase fused to affinity tags and fluorescent proteins used in the assay. (**B**) The pipeline of the fluorescent assay for the discovery of Ku70-IN inhibitors. On the first step, FP-tagged proteins are mixed with tested compounds in a pull-down buffer. On the second step, glutathione-coated beads are also added to the mix to precipitate GST-mCer-IN and GST-mCer-IN/His6-Ku70-tRFP. After washing of unprecipitated proteins (the third step) GST-mCer-IN and co-precipitated His6-Ku70-tRFP are eluted from beads under the mild denaturing conditions without heating to prevent loss of fluorescence of FP-tags. The level of mCer and tRFP can be assayed in plate-fluorometer or using gel-documentation station after separation of proteins in SDS-PAGE. The tRFP signal normalized to the mCer signal is used as a measure for inhibitors’ effectiveness. The additional points should be analyzed in the same experiment. One of them is a mix of all components except the tested compound. Another one is a mix, in which GST-mCer-IN is replaced by GST-mCer. The level of tRFP signal normalized to the mCer signal, in this case, is taken as the background.

For our system, we have chosen fluorescent proteins turboRFP (tRFP) and monomeric Cerulean (mCer). The choice of these particular proteins was based on three criteria: minimal overlap of excitation and emission spectra, which is necessary to prevent FP signal distortion due to Förster resonance energy transfer (FRET), relative brightness of both proteins, and absence of heterodimer formation between the two fluorescent proteins. When designing IN and Ku70 hybrids with fluorescent proteins, we considered previously obtained data indicating that binding of IN and Ku70 involves primarily the N-terminal domain of Ku70 (residues 1–250) and IN region from residue 200 to residue 220 [16]. To minimize the tags’ effect on the interaction between IN and Ku70, we have chosen to attach tRFP to the C-terminus of Ku70, and mCer–to the N-terminus of IN (Figure 1A). To do this, we cloned genes of the tRFP and mCer fluorescent proteins into vectors pET-15b-Ku70 and pGGWA-GST_IN and obtained bacterial expression vectors to produce His6-Ku70-tRFP and GST-mCer-IN, respectively. The hybrid proteins were extracted and purified by affinity chromatography on Ni-NTA-agarose and glutathione sepharose, respectively. 

**Figure 2:**
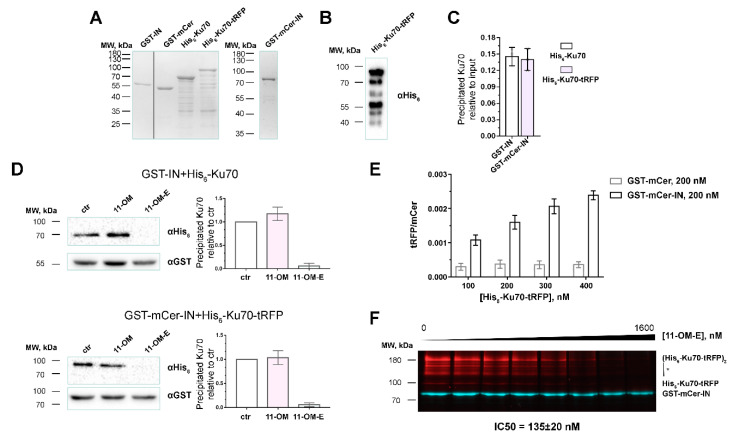
Validation of the fluorescent assay for the discovery of Ku70-IN inhibitors. (**A**) SDS-PAGE analysis of recombinant proteins GST-IN, GST-mCer, GST-mCer-IN, His_6_-Ku70, His_6_-Ku70-tRFP. (**B**) Western-blot analysis of His_6_-Ku70-tRFP purity using an anti-His_6_ antibody. (**C**) GST-mCer-IN (200 nM) binds His_6_-Ku70-tRFP (200 nM) at the same amount as GST-IN (200 nM) binds His_6_-Ku70 (200 nM). (**D**) Interaction of 200nM GST-IN and His_6_-Ku70 or GST-mCer-IN and His_6_-Ku70-tRFP in the absence or in the presence of well-characterized inhibitor 11-OM-E (1µM) or control compound 11-OM (1µM) is analyzed by GST-pull-down assay with subsequent WB analysis against GST and His_6_-tags. ctr–control, binding of proteins in absence of 11-OM-E or 11-OM. (**E**) Precipitation of His_6_-Ku70-tRFP with GST-mCer-IN or GST-mCer analyzed by the method described here in 96-well plate format. (**F**) Fluorescent pull-down assay analysis of the interaction of His_6_-Ku70-tRFP (200 nM) and GST-mCer-IN (200 nM) in the presence of an increasing concentration of 11-OM-E. * signed dimers of the full-length His_6_-Ku70-tRFP and N-terminal degraded forms.

Their purity was 35–45% for His_6_-Ku70-tRFP and 80–90% for GST-mCer-IN (Figure 2A). The purity of His_6_-Ku70-tRFP was comparable to that of His_6_-Ku70 (40–55% of the protein of interest), i.e. the introduction of FP did not significantly influence the stability of the protein. We had shown before that GST-Ku70 is subjected to proteolytic cleavage when expressed in *E. coli*, which leads to Ku70 preparations containing contaminants corresponding to the N-terminal fragments of the protein with the affine tag [16]. In the case of His_6_-Ku70-tRFP, we observed the same, i.e., the contaminants were detected with His_6_-tag antibodies (Figure 2B). Unfortunately, gel chromatography under non-denaturing conditions does not allow for the removal of N-terminal fragments from the preparations (data not shown), which may be explained by the dimerization of the N-terminal domains of Ku-70. 

To verify that the introduction of such massive tags as fluorescent proteins (mCer ~26.6 kDa and tRFP ~25.9 kDa) did not alter the structure of the proteins of interest IN and Ku70 and did not hinder their interaction due to steric shielding of the binding sites, we primarily estimated the possibility of complex formation between the obtained hybrid proteins and, for reference, between their original variants GST-IN and His_6_-Ku70. A pull-down assay showed that His_6_-Ku70-tRFP forms a complex with GST-mCer-IN at the same level as the non-modified proteins (Figure 2C). Although tRFP is known to form stable homodimers [38], this did not disrupt the system, what was confirmed by titration of GST-mCer-IN and GST-IN by His_6_-Ku70-tRFP and His_6_-Ku70, respectively (Supplementary Figure S2). 

In order to additionally confirm that the hybrid proteins interact similarly to the original IN and Ku70, we decided to use the previously described oligonucleotide inhibitor of their interaction. We had earlier found that IN catalytic activity inhibitor 11-OM-E hinders the interaction between IN and Ku70. Both the oligonucleotide moiety of the inhibitor and the eosin-Y fragment are important for this activity: free eosin-Y and an 11-OM-E analog without eosin-Y (11-OM) did not affect the stability of the IN/Ku70 complex [16]. We tested the effects of 11-OM-E and 11-OM on the pull-down of the non-modified (His_6_-Ku70 and GST-IN) and the FP-modified proteins (His_6_-Ku70-tRFP and GST-mCer-IN). Thus, 1 μM 11-OM-E turned out to completely prevent the interaction of the proteins of interest in both cases, whereas 11-OM exerted almost no effect on the coprecipitation of the proteins (Figure 2D), which additionally confirmed that the FP-tags did not influence binding of IN and Ku70.

The approach to the screening of inhibitors of binding of proteins with fluorescent tags suggested here may be used both in the high-throughput plate format and in the classical test-tube way with subsequent fluorescence analysis in the gel (Figure 1B). In the former case, a mixture of proteins and putative inhibitors is incubated with glutathione sepharose in 96-well spin-plates. After complex formation and the wash of the unbound proteins, GST-mCer-IN and GST-mCer-IN/Ku70-tRFP complexes are eluted from resin, and the signals from fluorescent proteins are measured. The tRFP/mCer ratio reflects the efficiency of complex formation. We assessed the applicability of this method to the analysis of coprecipitation of His_6_-Ku70-tRFP and GST-mCer-IN. As shown in Figure 2E, Ku70-tRFP is coprecipitated in a dose-dependent manner with GST-mCer-IN but not GST-mCer, which is a negative control confirming the workability of this approach. Of note, to simplify the test procedure we tried using glutathione-coated 96-well plates instead of glutathione sepharose and spin-plates. However, we did not manage to detect GST-mCer-IN immobilization in the plate or His_6_-Ku70-tRFP binding: the fluorescence levels of these proteins did not differ significantly from the background noise (data not shown), which can be explained by the insufficient binding capacity of such plates. 

When using glutathione sepharose and spin-plates, (Figure 1B), GST-mCer-IN and the GST-mCer-IN/Ku70-tRFP complex were separated in SDS-PAGE after elution from resin, and fluorescence was then detected in the gel. In this case, samples were not denatured at 94 °C as in standard sample preparation in order to maintain FP fluorescence [37]. Under conditions described, mCer and tRFP signals were linearly dependent on the amount of the proteins applied on the gel up to 40 pmol of proteins (the maximal tested amount, Supplementary Figure S3). However, due to the lack of the denaturing stage, His_6_-Ku70-tRFP produced several lines in the gel for it is prone to multimerization: the least intense line reflected the mobility of the monomer (~100 kDa), the brightest line corresponded to the (His_6_-Ku70-tRFP)2 dimer (>180 kDa) (Figure 2F). There was also a number of medium brightness lines, which might represent the dimers of a full-length His_6_-Ku70-tRFP with N-terminal fragments of the protein, which, as previously mentioned, were copurified with the full-length protein, (Figure 2F; 100 kDa < MW < 180 kDa, signed as *). Importantly, these lines were not present when samples were prepared in a standard manner including the denaturing stage and subsequent visualization using Coomasie G250 (Figure 2A). However, protein fluorescence levels estimation under denaturing conditions is impossible.

The gel electrophoresis stage decreases the productivity of the method, but it allows for studying inhibiting activity of fluorescent compounds. In particular, using SDS-PAGE we determined IC50 for previously characterized inhibitor 11-OM-E (Figure 2F). The IC50 value measured in our system equaled 135 ± 20 nM, which differed to a certain extent from a previously published value of 50 ± 10 nM [16]. This discrepancy may be due to the high sensitivity of IC50 to concentrations of proteins studied [39,40]. In the present work, to determine the inhibiting activity of 11-OM-E, we used both His_6_-Ku70-tRFP and GST-mCer-IN in 200 nM concentration, and in another research [16]–100 nM GST-Ku70 and 200 nM His_6_-IN. Consequently, the method presented here may be used to search for inhibitors as well as for the estimation of quantitative parameters of inhibition by compounds of interest.

### 3.2. The Effect of the Oligonucleotide Inhibitor Structure on Binding of IN and Ku70

11-OM-E inhibits both interaction of IN and Ku70 and catalytic activity of IN with comparable efficiencies [16,28]. The effect of the oligonucleotide inhibitor structure on IN activity has been extensively studied before [28]. We decided to use our assay system to test whether previous observations on the IN activity inhibition are valid for the disturbance of binding of IN and Ku70.

#### 3.2.1. Nucleobases Rather than the Sugar-Phosphate Backbone of the Inhibitor Are Crucial for IN Binding with Ku70

Primarily, using a series of 11-mer inhibitors with modifications in the sugar-phosphate backbone or the heterocyclic bases, we established structural motives in the oligonucleotide domain of the inhibitor necessary for the inhibition activity. As described above, we analyzed binding of His_6_-Ku70-tRFP to GST-mCer-IN in presence of increasing concentrations of 11-OM-E analogs using SDS-PAGE with subsequent fluorescence detection in gel.

Firstly, it turned out that substitution of 2′-O-methyl-oligonucleotide GGUUUUUGUGU (11-OM) with 2′-deoxyribooligonucleotide GGTTTTTGTGT (11-D) in the eosin conjugate did not affect inhibiting activity: the IC50 values of 11-OM-E and 11-D-E in our test were almost equal (IC50 = 135 ± 20 nM vs. 150 ± 30). Secondly, we found that decrease in charge of the 2′-deoxyribooligonucleotide by a substitution of phosphodiester groups with methylphosphonates did not affect the inhibition potential of the corresponding conjugates (11-D-E, 11-DX-E-1 and 11-DX-E-2; Table 1). Thirdly, an alteration of the hydrophobic properties of the sugar-phosphate backbone and charge delocalization on a more massive sulfur atom resulted from the substitution of phosphodiester groups with phosphorothioates increased inhibitory potential 3.75-fold (11-DS-E; Table 1).

We have also analyzed an effect of nucleic base elimination on the inhibiting properties of conjugates. Because of some specific features of synthesis of such derivatives, a dye was decided to be introduced during the synthesis at the 5′-end of the oligonucleotide moiety, which is complicated for eosin-Y. It had been demonstrated before that a substitution of eosin-Y with 6-carboxy-4,7,2′,4′,5′,7′-hexachlorofluorescein (HEX) in the conjugates and a translocation of the hydrophobic moiety from the 3’-end to the 5′-end do not affect their ability to suppress IN catalytic activity [28]. To make sure that these alterations in the structure of the inhibitor would not affect its ability to impair IN/Ku70 complex formation, we synthesized Hex-11-D and tested its inhibiting properties. In our system, Hex-11-D demonstrated IC50 values comparable to those of 11-OM-E and 11-D-E (Table 1). After that, we prepared a Hex-11-D analog–Hex-11-ddR–lacking nucleic bases at 1–10 positions from 5′-end. Hex-11-ddR did not affect the stability of the IN/Ku70 complex up to 3.2 μM (Table 1). This result shows that nucleobases are critical for these compounds’ capacity to hinder IN/Ku70 complex formation.

#### 3.2.2. The Inhibition Efficiency of the Eosin-Y-Conjugates Depends on the Length of Their Oligonucleotide Moiety

**Figure 3:**
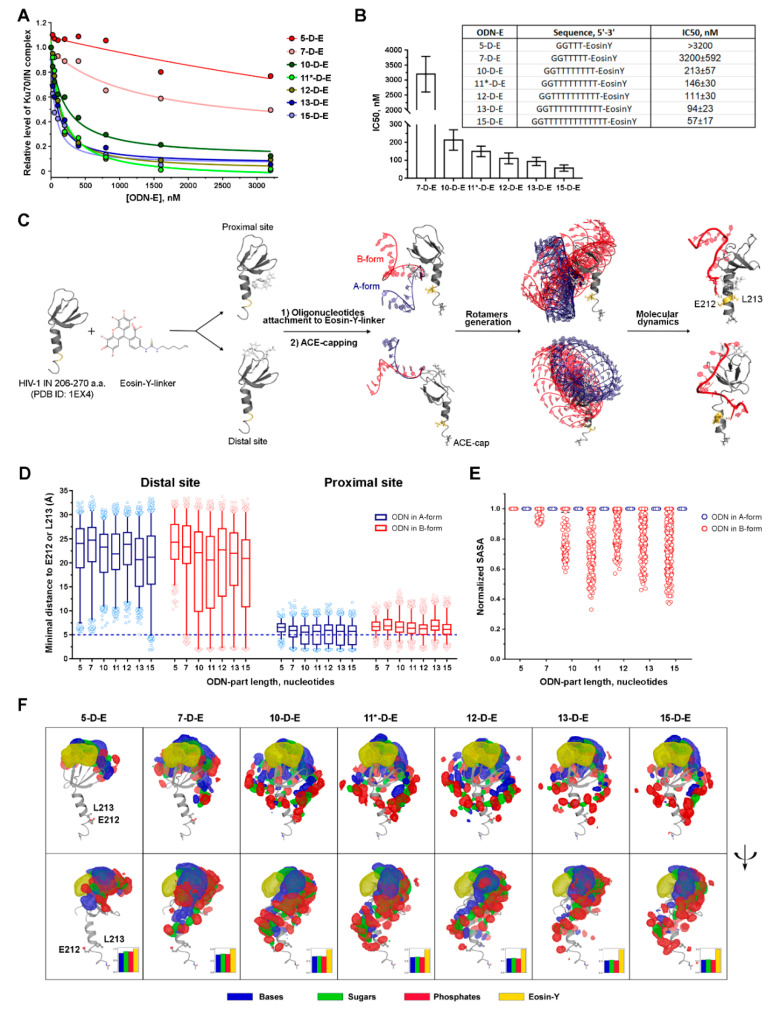
An effect of the oligodeoxynucleotide length of ODN-E on the complex formation between the Ku70 and HIV-1 integrase. (**A**) Fluorescent pull-down assay analysis of the interaction of His_6_-Ku70-tRFP (200 nM) and GST-mCer-IN (200 nM) in presence of an increasing concentration of ODN-E with different size of ODN part (5–15 nucleotides). (**B**) ODN-E sequences and IC50 values measured in a fluorescent pull-down assay. (**C**) The pipeline of the molecular dynamics experiments for IN/ODN-E systems. Eosin-Y+linker was docked to the HIV-1 IN (PDB ID 1EX4, residues 206–270) and two complexes with Eosin-Y+linker bound at proximal and distal sites were obtained. After that, DNA oligonucleotides in either A or B forms were attached to the Eosin-Y+linker. To prevent the undesirable interactions between ODN-E and N-terminal charged amino group ACE-cap (acetyl group) was added to the N-terminus. The molecular dynamics simulations were carried out after the rotamers generation as described in the Materials and Methods section. (**D**) Distribution of minimal distances between atoms of the ODN-E and the E212/L213 residues of IN for different starting systems: eosin-Y bound at a proximal or distal site in C-terminal domain of IN, A- or B-ODN forms. (**E**) Distribution of normalized solvent-accessible surface area (SASA) of E212/L213 in complexes of HIV-1 IN with ODN-E obtained in the molecular dynamics simulations for starting systems with eosin-Y bound at the distal site and ODN in A- or B-form. (**F**) Density maps of the ODN-E. Atom positions belonging to the particular fragments (eosin-Y, sugars, phosphates, bases) were averaged across all oligonucleotide structures and depicted with meshes at the 3σ level. Atoms, including hydrogens, were treated as spheres with the vdW radius of corresponding chemical elements. Phosphates are colored in red, sugars in green, bases in blue, and eosin-Y in gold.

We suggested before, based on experimental data, that 11-OM-E may act as a competitive inhibitor of IN/Ku70 complex formation by shielding residues of IN involved in the interaction with Ku70, namely, E212 and L213 [16]. To test this hypothesis, we decided to estimate the effect of the length of inhibitor oligonucleotide moiety on inhibition efficiency. A series of conjugates of 2′-oligodeoxyribonucleotides GG(T)n (n = 3, 5, 8, 9, 10, 11 и 13) was obtained, which we named 5-D-E, 7-D-E, 10-D-E, 11*-D-E, 12-D-E, 13-D-E, and 15-D-E respectively. IC50 values were determined for these compounds using our test system. First of all, it is noteworthy that a G→T heterocyclic base substitution in the 3′-end of the 11-mer oligonucleotide did not affect inhibition properties of the conjugate: the IC50 values turned out to be equal for 11-D-E and 11*-D-E (Table 1 and Figure 3B). As shown in Figure 3A,B, the inhibitory efficiency of the conjugates decreased with decrease in the oligonucleotide length. While the IC50 value of 10-D-E equaled 213 ± 57 nM, which was insignificantly higher than that of 11*-D-E, 7-D-E hinders IN/Ku70 complex formation by 50% in the maximal inhibitor concentration tested (3.2 μM), and 5-D-E–by only 24% under the same conditions. Accordingly, increase in length of the inhibitor oligonucleotide moiety (12-D-E, 13-D-E, 15-D-E) caused increase in the inhibitors’ efficiency, which manifested in IC50 value decrease (Figure 3A,B). However, this effect was not so important. Comparing the IC50 values of 11*-D-E vs. 15-D-E (a 2.56-fold decrease in IC50) and 7-D-E vs. 11-D*-E (a more than 20-fold decrease in IC50) revealed that the impact of the first 10–11 nucleotides was much higher than that of subsequent ones (12–15). Therefore, the 11-mer conjugate sufficed to inhibit IN/Ku70 complex formation.

### 3.3. The Effect of the Oligonucleotide Moiety Structure on the Conjugates’ Inhibition Potential May Be Due to the Steric Shielding of the Integrase Surface Involved in the Complex Formation

According to the experiment, as the length of the oligonucleotide decreases, the inhibitory activity of the conjugates decreases as well. This effect may occur due to the physical shielding of the 212/213 amino acids of IN by the oligonucleotide. We hypothesized that short oligonucleotides are limited in the ability to perform such shielding. To verify this hypothesis, we performed molecular modeling of IN in complex with oligodeoxyribonucleotides of different length conjugated to eosin-Y (ODN-E). As the inhibitors in our study are long flexible polymers, it is hard to expect one particular complex conformation. However, it is possible to estimate the repertoire of the possible positions of the inhibitors using molecular dynamics approaches. We suggested before, based on experimental data, that 11-OM-E may act as a competitive inhibitor of IN/Ku70 complex formation: eosin serves as an anchor, being fastened in a certain position by hydrophobic contacts with IN while an oligonucleotide moiety reaches and shields residues of IN involved in the interaction with Ku70, namely, E212 and L213 [16]. A series of structures of IN complexes with inhibitors of various lengths (5-D-E–15-D-E) was obtained by adding DNA oligonucleotides in A- or B-form to eosin-Y+linker moiety bound in two sites identified previously and called proximal and distal due to their distance from E212 and L213 a.a. of IN [16]. The structures were then subjected to molecular modeling (Figure 3C).

The shielding of E212/L213 may occur due to a direct interaction with an oligonucleotide or due to a conformational change in an α6-helix that makes the E212/L213 less accessible for Ku70. To consider both possibilities, we separately analyzed a distance between the inhibitor and selected amino acids and a solvent-accessible surface area (SASA) for E212/L213 as shielding criteria (Figure 3D,E). For the distal and the proximal sites, we observed principally different dependencies of a minimal distance between the inhibitor and E212/L213 on the length of oligonucleotide. 

When eosin-Y was anchored in the proximal site, the distributions of the minimal distance do not significantly depend on the length of the oligonucleotide moiety of the inhibitor. For each inhibitor, there was a series of complexes where ODN-E interacted with the selected amino acids (Figure 3C, within 5 Å). Therefore, the proximal site model did not correspond to the experimental data and, probably, is not utilized in the reality. Compounds with eosin bound in the distal site, on the contrary, reached the threshold of contact distance (5 Å) starting from 7-D-E, which corresponded to the experimental IC50 (Figure 3D). This relation is observed for initial systems with the oligonucleotide moiety in B-form, but not in A-form (Figure 3D,F), although previously we had observed the distances shorter than 5 Å for 11-OM-E in A-form even for the distal site [16]. The difference in the distribution of distances from inhibitor to E212/L213 a.a. for 11-OM-E [16] and 11*-D-E both started from the A-form structures must be due to greater flexibility of ssDNA comparing to ssRNA, which is even more pronounced near polar surfaces, such as proteins [28]. Moreover, in the case of 2′-O-Me-RNA, flexibility and thus a possible conformational landscape is even lower due to the steric hindrances imposed by the presence of the methyl group.

For the inhibitors anchored in the distal site, we also analyzed SASA for E212/L213 a.a. Since absolute values of SASA are not informative, a distribution of relative SASA values for E212/L213 normalized to SASA values for complexes with conjugates excluded from structures was analyzed (Figure 3E). The ratio less than 1 was observed for oligonucleotides of length 7 and more, which means that 5-D-E did not affect the solvent-accessible surface area around E212/L213. This relation was observed for initial systems with ODN in B-form. In the case of A-form, all the relative SASA values equaled 1 (Figure 3E). Similar effects can be seen on density maps of ODN-E in complex with IN (Figure 3F). Importantly, the observed SASA values around E212/L213 in structures with the inhibitor extracted were independent of the inhibitor’s length (Supplementary Figure S4A), which implies that the impact of conformational rearrangements in the shielding was inconsiderable. At the same time, when the inhibitor was present, notable decrease in SASA values was seen (Supplementary Figure S4B). This effect is consistent with length dependency observed for distances. 

Minimal distances and SASA analyses suggest that oligonucleotide conjugates with eosin-Y can shield E212/L213 of HIV-1 IN in a length-dependent manner, which confirms the hypothesized mechanism of action for these inhibitors described above. 

Furthermore, we analyzed interaction of different building blocks of 11*-D-E (phosphates, sugars, bases and eosin-Y) with IN to decipher their role in its inhibiting activity. Higher frequency of interaction of nucleic bases and sugars rather than phosphates of 11*-D-E with IN was detected (Supplementary Figure S5). Interestingly, only bases were found to contact with amino acids from 206 to 212 located in the α6-helix of IN. Since the removal of nucleic bases from the inhibitor sharply reduced its activity, it is possible that these contacts are crucial for the efficient shielding E212/L213a.a.

## 4. Discussion

Just 20–30 years ago, PPIs were considered as “undruggable” targets. However, a growing number of positive results of using low-molecular compounds to modulate cellular functions by affecting PPI has caused the necessity to reconsider this concept [41]. PPI inhibitors are considered promising for curing various human virus diseases, such as HIV-infection. HIV-1 uses a variety of cellular proteins, the interaction of which with those of the virus is critical for successful replication. While using classical inhibitors of viral enzymes is associated with resistance development in the case of RNA-containing viruses, using PPI inhibitors aimed at cellular and viral proteins interaction disruption should exert no such harmful effects [42]. Indeed, such inhibitors are already being designed. As previously noted, the most progress in HIV-1 studies is made for the complex of HIV-1 integrase and cellular protein- LEDGF/p75, for which complex structures have been determined [12].

We have recently characterized interaction of HIV-1 IN with another cellular partner–Ku70 [16,17], the interaction with which had been shown before [15]. In particular, a functional role of this interaction in post-integration DNA repair has been shown [17] and IN residues involved in complex formation have been determined [16]. We suppose that HIV-1 IN-Ku70 complex should be regarded as a novel promising target for anti-HIV drugs. In the present study, we have designed a fluorescence-based method of search for inhibitors of the interaction of IN and Ku70, which is a modified FluorIA method [26]. We have shown that this method can be used to assess efficiency of interaction of Ku70 and IN in a plate format, as well as to study effects of inhibitors with proper fluorescence in the same channels as the proteins of interest in an SDS-PAGE format. The latter variant is possible due to the low sensitivity of some fluorescent proteins to SDS in the absence of heating [37]. Indeed, in our experiment, mCer and tRFP signals were linearly correlated with the amount of the proteins applied to the gel (Supplementary Figure S3). The system has been validated using 11-OM-E, the only known inhibitor of interaction of IN and Ku70 we had described before, and 11-OM, its analog displaying no inhibiting activity [16].

In the present study, we used a series of derivatives of 11-OM-E and studied dependence of the inhibiting effect exerted on the IN/Ku70 complex on the structure of these compounds. We had previously hypothesized a competitive mechanism of inhibition of IN/Ku70 complex formation by 11-OM-E. It was also suggested that the oligonucleotide moiety of the inhibitor might shield amino acid residues E212 and L213 in IN, which are critical for its interaction with Ku70, while eosin serves as an anchor, being fastened in a certain position by hydrophobic contacts with IN. Here, using our analytical system, we have experimentally tested the effect of length alterations of the oligonucleotide moiety of the conjugate (5–15-mer) on its inhibiting activity and have performed additional in silico experiments to model structures of IN complexes with inhibitors of various lengths in compliance with a previously published protocol [16]. In total, our data support the suggestion that the inhibition of IN/Ku70 complex formation may be mediated by shielding of IN residues E212 and L213, which are crucial for its interaction with Ku70. Moreover, the in-silico experiments show that these amino acids are shielded when eosin is anchored in the distal site in the C-terminal domain of IN.

The shielding may be mediated by immediate contacts between IN and the sugar-phosphate backbone or the nucleic bases of the inhibitor. To clarify it, we also characterized the effects of inhibitors with modifications in the sugar-phosphate backbone or lacking some nucleic bases. We found that a substitution of 2′-O-methylated ribose with 2′-deoxyribose does not affect the inhibition efficiency. The elimination of three negative charges in the sugar-phosphate backbone did not significantly affect the inhibiting activity either, whereas a substitution of oxygen atoms with sulfur increased the inhibiting activity four-fold. This could be explained both by increased localization of the negative charge on the sulfur atoms when compared to oxygens in phosphodiesters, and by higher hydrophobic properties of phosphorothioates than those of phosphodiesters. The most pronounced effect of oligonucleotide moiety modifications was observed with an elimination of 10 bases of 11 from the inhibitor. Apparently, an appropriate folding of the inhibitor on the surface of IN necessary for its inhibiting properties results from contacts between amino acids in region of IN from 206 to 212 a.a. and nucleic bases rather than the sugar-phosphate backbone (Supplementary Figure S5), although the backbone may also have a certain impact, which is observed in the case of phosphorothioates. It is also important to stress that the presence of nucleic bases rather their sequence is critical for the inhibiting activity. This finding as well as the inhibitory activity increase with the O→S substitution suggest that the appropriate folding is based primarily on hydrophobic interactions.

Of note, such effects, including inhibiting activity not being dependent on oligonucleotide sequence, had previously been observed for impact of oligonucleotide inhibitors on IN catalytic activity [27,43,44]. Interestingly, these inhibitors are able to destroy the already formed complex of IN and DNA [44], and we had not managed to explain this property before. It is clear now that the inhibitor uses its hydrophobic aromatic part (eosin) to bind to the C-terminal domain of IN and its oligonucleotide moiety to shield the surface of the α6-helix, which links the C-terminal and the catalytic domains. Moreover, this binding can result in a certain structural deformation of the α6-helix [16]. Taking into account that it is the C-terminal and catalytic domains that bind viral DNA [45,46], and the α6-helix interacts with Ku70, it becomes clear why similar structural alterations of the inhibitor have similar effects on the inhibition of both the catalytic activity of IN and its binding to Ku70.

## 5. Conclusions

Summing up, we have managed to design a simple system to analyze interaction of HIV-1 IN and human protein Ku70, and have successfully used this system to characterize a structural basis of the impairment of these proteins’ interaction by an oligonucleotide conjugate with eosin. These data should be taken into account in the further in silico search for novel inhibitors of this interaction.

## Figures and Tables

**Table 1:**
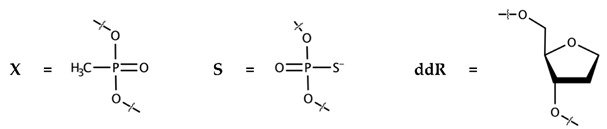
Modifications of the oligonucleotide structure and their effects on the inhibition of the Ku70/IN complex formation.

ODN-E	Sequence, 5′-3′	IC50, nM
11-OM-E	GGUUUUUGUGU-EosinY	135 ± 20
11-D-E	GGTTTTTGTGT-EosinY	150 ± 30
11-DX-E-1	GGTTTTTxGTxGTx-EosinY	160 ± 55
11-DX-E-2	GGTxTxTxTTGTGT-EosinY	150 ± 40
11-DS-E	GsGsTsTsTsTsTsGsTsGsTs-EosinY	40 ± 10
Hex-11-D	HEX-GGTTTTTGTGT	170 ± 45
Hex-11-ddR	HEX-(ddR)_10_T	No inhibition up to 3200 nM

## Supplementary Materials

The following are available online at https://www.mdpi.com/2218-273X/10/9/1236/s1. Figure S1. Sampling estimation map. For each rotamer of each structure, we estimated the number of replicas sufficient to observe distances between 212/213 a.a. and an oligonucleotide close to the best possible. The numbers on the map are the estimates. They were calculated as follows: after a modeling in 100 repetitions, a distance between any atom of an oligonucleotide and 212 or 213 amino acid was calculated for every repetition of every rotamer. After that, from 100 distances for every rotamer, we took 1000 independent samples of size n with repetitions, took a minimum for each sample and calculated how many m minimums (out of 1000) were less than or equal to 5 Å, rounded up minimal distance among initial 100 replicates, which was a criteria of proximity to minimum. A size n of samples that gave us m ≥950 out of 1000 minimums close to the global minimum was assumed sufficient and placed on the map. If the estimated value was >100, the cell was left blank. Structures on the map were surrounded with a blue frame if a distance less or equal to 5A was observed among 100 repetitions. These structures are the most interesting since their initial position allows them to get close to the target amino acids, while others can be originally oriented so that their interaction with the protein is not sufficient. Figure S2. GST-pull down assay analysis of the binding of His_6_-Ku70-tRFP or His_6_-Ku70 with GST-mCer-IN or GST-IN, respectively. Concentration of the GST-mCer-IN and GST-IN was 50 nM. Figure S3. Dependence of mCer and tRFP fluorescent signal measured in gel on amount of GST-mCer-IN (**a**) and His6-Ku70-tRFP (**b**). Figure S4. Distribution of solvent-accessible surface area (SASA) of E212/L213 a.a. with oligonucleotide inhibitors are being extracted from the complexes of HIV-1 IN with ODN-E obtained in molecular dynamic experiments (**a**) or with inhibitors (**b**). Only systems with eosin-Y bound at distal site and ODN in A- or B-forms were considered. Figure S5. Cumulative contacts between IN and oligonucleotide atoms of 11*-D-E. Each data point represents the total number of contacts (defined as a distance less or equal than 5 Å) between particular protein atom and all oligonucleotide atom normalized by the total number of oligonucleotide configurations. Phosphates are colored in red, sugars in green, bases in blue, and eosin-Y in gold. Table S1. MALDI MS analysis of the oligonucleotides used in the study.

## References

[1] Deeks S.G., Lewin S.R., Havlir D.V. (2013). The end of AIDS: HIV infection as a chronic disease. Lancet.

[2] Kuritzkes D.R. (2011). Drug resistance in HIV-1. Curr. Opin. Virol..

[3] Pennings P.S. (2013). HIV drug resistance: Problems and perspectives. Infect. Dis. Rep..

[4] Günthard H.F., Calvez V., Paredes R., Pillay D., Shafer R.W., Wensing A.M., Jacobsen D.M., Richman D.D., Francisco S. (2018). Human Immunodeficiency Virus Drug Resistance: 2018 Recommendations of the International Antiviral Society-USA Panel and 8 International Antiviral Society-USA. HIV Drug Resist. Recomm. CID.

[5] Gupta R.K., Gregson J., Parkin N., Haile-Selassie H., Tanuri A., Andrade Forero L., Kaleebu P., Watera C., Aghokeng A., Mutenda N. (2018). HIV-1 drug resistance before initiation or re-initiation of first-line antiretroviral therapy in low-income and middle-income countries: A systematic review and meta-regression analysis. Lancet Infect. Dis..

[6] Taltynov O., Desimmie B.A., Demeulemeester J., Christ F., Debyser Z. (2012). Cellular Cofactors of Lentiviral Integrase: From Target Validation to Drug Discovery. Mol. Biol. Int..

[7] Taltynov O., De Rijck J., Debyser Z. (2018). Identification and Validation of HIV Cofactors. Encyclopedia of AIDS.

[8] Dürr R., Keppler O., Christ F., Crespan E., Garbelli A., Maga G., Dietrich U. (2015). Targeting cellular cofactors in HIV therapy. Top. Med. Chem..

[9] Poeschla E.M. (2008). Integrase, LEDGF/p75 and HIV replication. Cell. Mol. Life Sci..

[10] Christ F., Debyser Z. (2013). The LEDGF/p75 integrase interaction, a novel target for anti-HIV therapy. Virology.

[11] Le Rouzic E., Bonnard D., Chasset S., Bruneau J.M., Chevreuil F., Le Strat F., Nguyen J., Beauvoir R., Amadori C., Brias J. (2013). Dual inhibition of HIV-1 replication by integrase-LEDGF allosteric inhibitors is predominant at the post-integration stage. Retrovirology.

[12] Christ F., Voet A., Marchand A., Nicolet S., Desimmie B.A., Marchand D., Bardiot D., Van Der Veken N.J., Van Remoortel B., Strelkov S.V. (2010). Rational design of small-molecule inhibitors of the LEDGF/p75-integrase interaction and HIV replication. Nat. Chem. Biol..

[13] Kessl J.J., Kutluay S.B., Townsend D., Rebensburg S., Slaughter A., Larue R.C., Shkriabai N., Bakouche N., Fuchs J.R., Bieniasz P.D. (2016). HIV-1 Integrase Binds the Viral RNA Genome and Is Essential during Virion Morphogenesis. Cell.

[14] Choi E., Mallareddy J.R., Lu D., Kolluru S. (2018). Recent advances in the discovery of small-molecule inhibitors of HIV-1 integrase. Future Sci. OA.

[15] Zheng Y., Ao Z., Wang B., Jayappa K.D., Yao X. (2011). Host protein Ku70 binds and protects HIV-1 integrase from proteasomal degradation and is required for HIV replication. J. Biol. Chem..

[16] Anisenko A.N., Knyazhanskaya E.S., Zalevsky A.O., Agapkina J.Y., Sizov A.I., Zatsepin T.S., Gottikh M.B. (2017). Characterization of HIV-1 integrase interaction with human Ku70 protein and initial implications for drug targeting. Sci. Rep..

[17] Knyazhanskaya E., Anisenko A., Shadrina O., Kalinina A., Zatsepin T., Zalevsky A., Mazurov D., Gottikh M. (2019). NHEJ pathway is involved in post-integrational DNA repair due to Ku70 binding to HIV-1 integrase. Retrovirology.

[18] Blackford A.N., Jackson S.P. (2017). ATM, ATR, and DNA-PK: The Trinity at the Heart of the DNA Damage Response. Mol. Cell.

[19] Mohiuddin I.S., Kang M.H. (2019). DNA-PK as an Emerging Therapeutic Target in Cancer. Front. Oncol..

[20] Rao V.S., Srinivas K., Sujini G.N., Sunand Kumar G.N. (2014). Protein-Protein Interaction Detection: Methods and Analysis. Int. J. Proteom..

[21] Eglen R.M., Reisine T., Roby P., Rouleau N., Illy C., Bossé R., Bielefeld M. (2008). The Use of AlphaScreen Technology in HTS: Current Status. Curr. Chem. Genom..

[22] Ossiboff R.J., Zhou Y., Lightfoot P.J., Prasad B.V.V., Parker J.S.L. (2010). Conformational Changes in the Capsid of a Calicivirus upon Interaction with Its Functional Receptor. J. Virol..

[23] Weng Z., Zhao Q. (2015). Utilizing ELISA to monitor protein-protein interaction. Protein-Protein Interactions: Methods and Applications.

[24] Pinto M.G.V., Baiker A. (2012). LuMPIS: Luciferase-based MBP-Pull-down protein interaction screening system. Methods Mol. Biol..

[25] Bacart J., Corbel C., Jockers R., Bach S., Couturier C. (2008). The BRET technology and its application to screening assays. Biotechnol. J..

[26] Al-Mugotir M., Kolar C., Vance K., Kelly D.L., Natarajan A., Borgstahl G.E.O. (2019). A simple fluorescent assay for the discovery of protein-protein interaction inhibitors. Anal. Biochem..

[27] Prikazchikova T.A., Volkov E.M., Zubin E.M., Romanova E.A., Gottikh M.B. (2007). Inhibition of HIV-1 integrase by modified oligonucleotides: Optimization of the inhibitor structure. Mol. Biol..

[28] Agapkina J., Zatsepin T., Knyazhanskaya E., Mouscadet J.F., Gottikh M. (2011). Structure-activity relationship studies of HIV-1 integrase oligonucleotide inhibitors. ACS Med. Chem. Lett..

[29] Anisenko A.N., Knyazhanskaya E.S., Zatsepin T.S., Gottikh M.B. (2017). Human Ku70 protein binds hairpin RNA and double stranded DNA through two different sites. Biochimie.

[30] Knyazhanskaya E.S., Smolov M.A., Kondrashina O.V., Gottikh M.B. (2009). Relative Comparison of Catalytic Characteristics of Human Foamy Virus and HIV-1 Integrases. Acta Nat..

[31] Li S., Olson W.K., Lu X.-J. (2019). Web 3DNA 2.0 for the analysis, visualization, and modeling of 3D nucleic acid structures. Web Serv. Issue Publ. Online.

[32] Abraham M.J., Murtola T., Schulz R., Páll S., Smith J.C., Hess B., Lindah E. (2015). Gromacs: High performance molecular simulations through multi-level parallelism from laptops to supercomputers. SoftwareX.

[33] Bakan A., Meireles L.M., Bahar I. (2011). ProDy: Protein Dynamics Inferred from Theory and Experiments. Bioinforma. Appl. NOTE.

[34] Gil V.A., Guallar V. (2013). pyRMSD: A Python package for efficient pairwise RMSD matrix calculation and handling. Bioinformatics.

[35] Team T. (2020). Pandas development Pandas-dev/pandas: Pandas. Zenodo.

[36] Hunter J.D. (2007). Matplotlib: A 2D graphics environment. Comput. Sci. Eng..

[37] Saeed I.A., Ashraf S.S. (2009). Denaturation studies reveal significant differences between GFP and blue fluorescent protein. Int. J. Biol. Macromol..

[38] Shcherbo D., Merzlyak E.M., Chepurnykh T.V., Fradkov A.F., Ermakova G.V., Solovieva E.A., Lukyanov K.A., Bogdanova E.A., Zaraisky A.G., Lukyanov S. (2007). Bright far-red fluorescent protein for whole-body imaging. Nat. Methods.

[39] Cer R.Z., Mudunuri U., Stephens R., Lebeda F.J. (2009). *IC*_50_-to-*K*_i_: A web-based tool for converting *IC*_50_ to *K*_i_ values for inhibitors of enzyme activity and ligand binding. Nucleic Acids Res..

[40] Kalliokoski T., Kramer C., Vulpetti A., Gedeck P. (2013). Comparability of Mixed IC50 Data–A Statistical Analysis. PLoS ONE.

[41] Guy A.T., Piggot T.J., Khalid S. (2012). Single-stranded DNA within nanopores: Conformational dynamics and implications for sequencing; A molecular dynamics simulation study. Biophys. J..

[42] Ran X., Gestwicki J.E. (2018). Inhibitors of protein–protein interactions (PPIs): An analysis of scaffold choices and buried surface area. Curr. Opin. Chem. Biol..

[43] Benoıˆt De Chassey B., Ne Meyniel-Schicklin L., Aublin-Gex A., André P., Lotteau V. (2012). New horizons for antiviral drug discovery from virus-host protein interaction networks. Curr. Opin. Virol..

[44] Pinskaya M., Romanova E., Volkov E., Deprez E., Leh H., Brochon J.C., Mouscadet J.F., Gottikh M. (2004). HIV-1 integrase complexes with DNA dissociate in the presence of short oligonucleotides conjugated to acridine. Biochemistry.

[45] Chen J.C.H., Krucinski J., Miercke L.J.W., Finer-Moore J.S., Tang A.H., Leavitt A.D., Stroud R.M. (2000). Crystal structure of the HIV-1 integrase catalytic core and C-terminal domains: A model for viral DNA binding. Proc. Natl. Acad. Sci. USA.

[46] Lutzke R.A.P., Vink C., Plasterk R.H.A. (1994). Characterization of the Minimal DNA-Binding Domain of the HIV Integrase Protein. Nucleic Acids Res..

