# Supplementary material for: A Fluorescent Assay to Search for Inhibitors of HIV-1 Integrase Interactions with Human Ku70 Protein, and Its Application for Characterization of Oligonucleotide Inhibitors
Source: Biomolecules. 2020 Aug 25;10(9):1236. doi: 10.3390/biom10091236 (PMC7563236; doi:10.3390/biom10091236)
Supplement: Supplementary file 1 [file biomolecules-10-01236-s001.zip › Table S1.docx]

**Table S1.** MALDI MS analysis of the modified oligonucleotides used in the study. MALDI MS spectra were registered on AutoFlex (Bruker Daltonics) using 2,4,6-trihydroxyacetophenone/ ammonium cirtrate or 3- hydroxypicolinic acid/ ammonium cirtrate as a matrix.

| **Conugate** | **Calculated mass, M+H+** | **Found mass, M+H+** |
| --- | --- | --- |
| 11-OM-E | 4501.4 | 4501.6 |
| 11-D-E | 4091.2 | 4091.4 |
| 11-DX-E-1 | 4263.4 | 4264.2 |
| 11-DX-E-2 | 4263.4 | 4263.9 |
| 11-DS-E | 4430.0 | 4432.9 |
| Hex-11-D | 4129.4 | 4130.2 |
| Hex-11-ddR | 2788.3 | 2788.6 |
| 5-D-E | 2216.0 | 2216.5 |
| 7-D-E | 2824.3 | 2824.4 |
| 10-D-E | 3736.9 | 3737.7 |
| 11*-D-E | 4041.1 | 4041.8 |
| 12-D-E | 4345.3 | 4345.5 |
| 13-D-E | 4649.5 | 4650.2 |
| 15-D-E | 5257.9 | 5258.7 |
