# Supplementary figures and images for: A Fluorescent Assay to Search for Inhibitors of HIV-1 Integrase Interactions with Human Ku70 Protein, and Its Application for Characterization of Oligonucleotide Inhibitors
Source: Biomolecules. 2020 Aug 25;10(9):1236. doi: 10.3390/biom10091236 (PMC7563236; doi:10.3390/biom10091236)

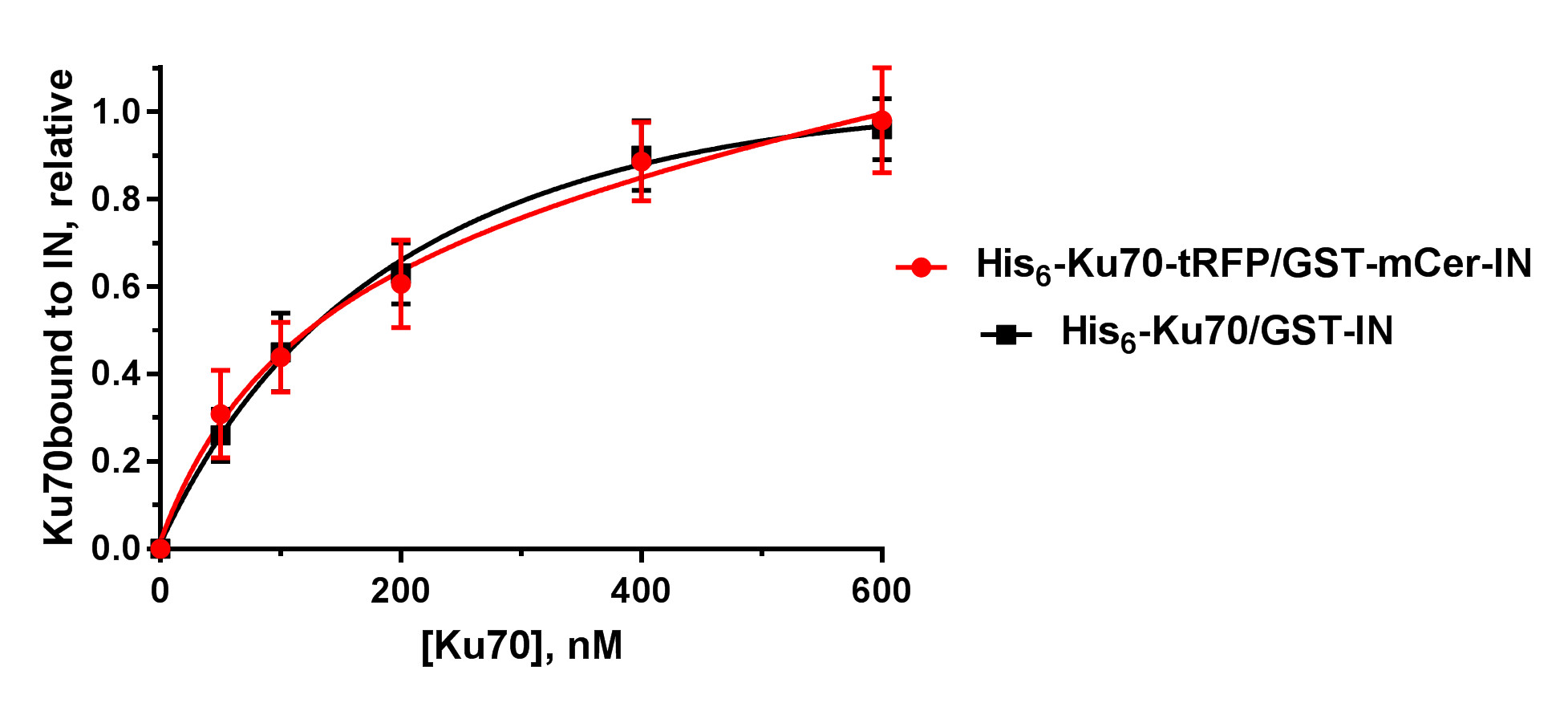

Supplement: Supplementary file 1 [file biomolecules-10-01236-s001.zip › Fig_S2.tif]

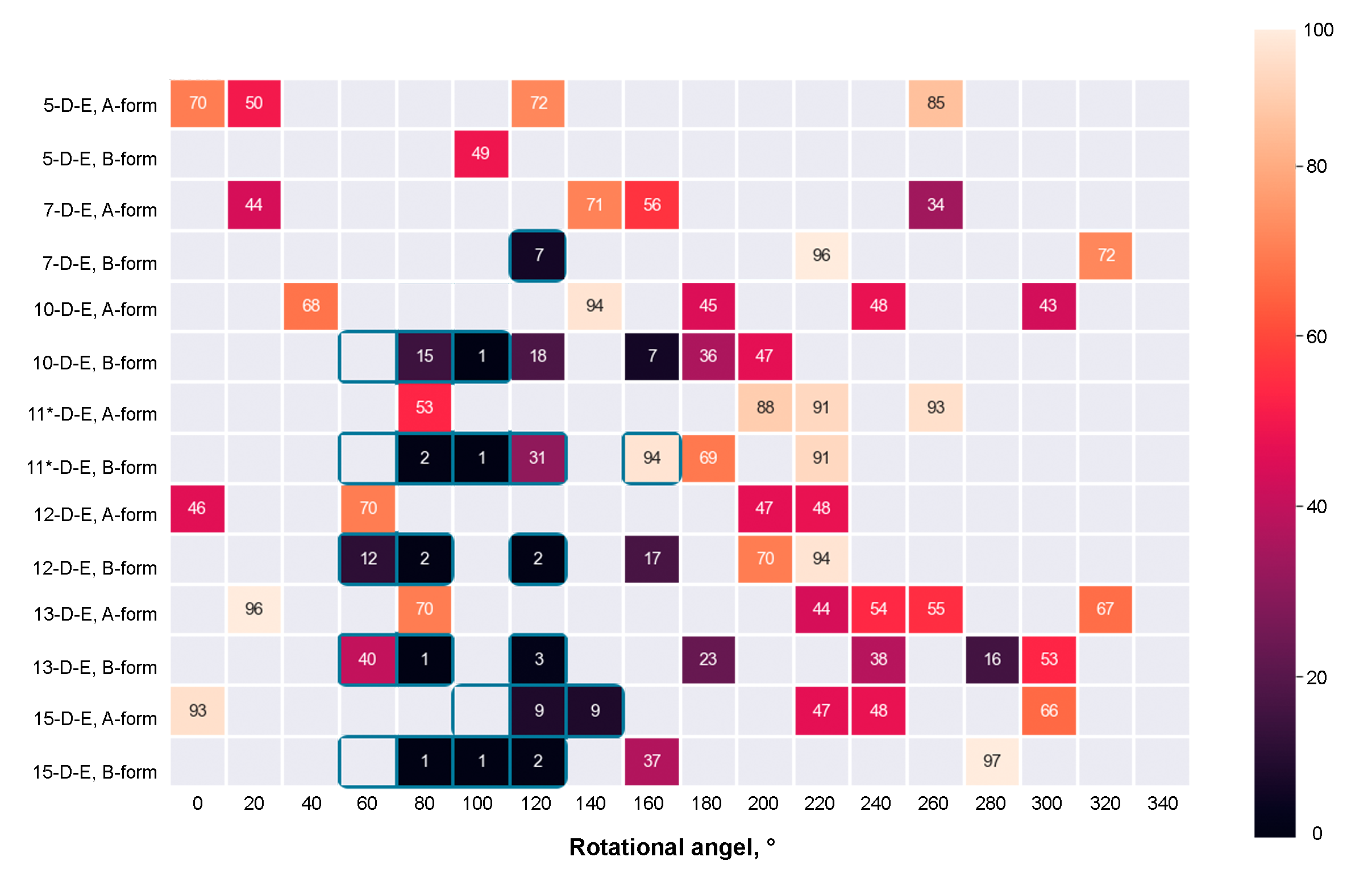

Supplement: Supplementary file 1 [file biomolecules-10-01236-s001.zip › Fig_s1.tif]

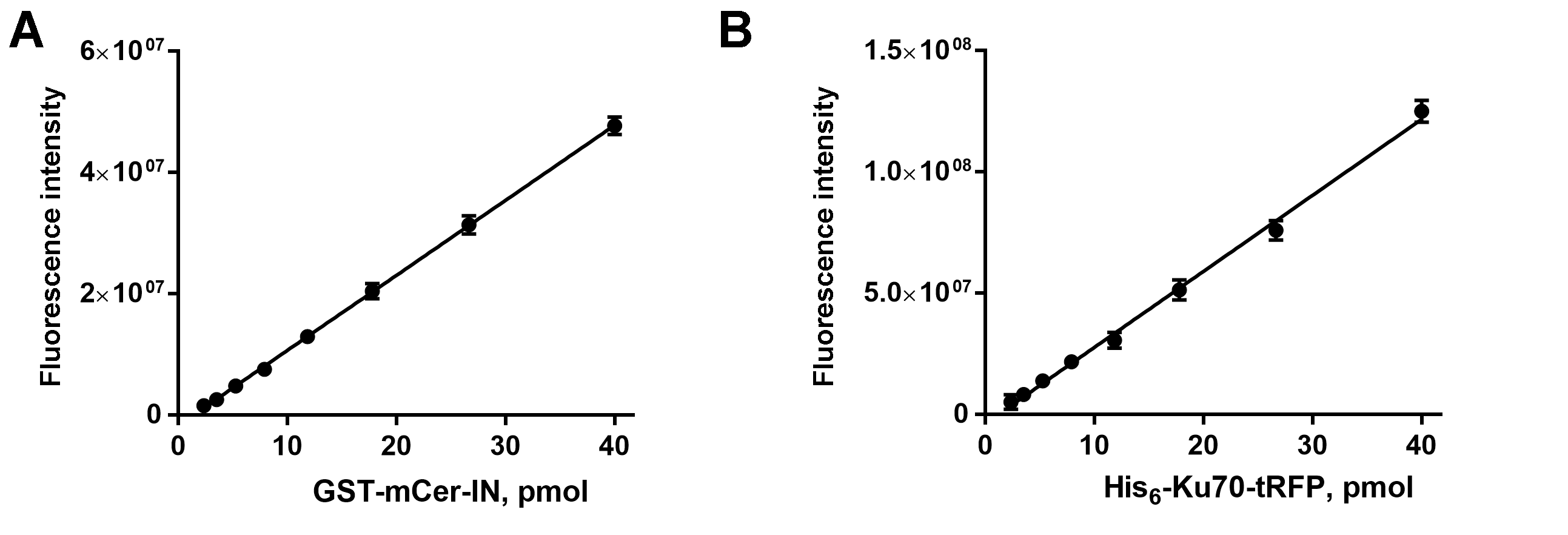

Supplement: Supplementary file 1 [file biomolecules-10-01236-s001.zip › Fig_s3.tif]

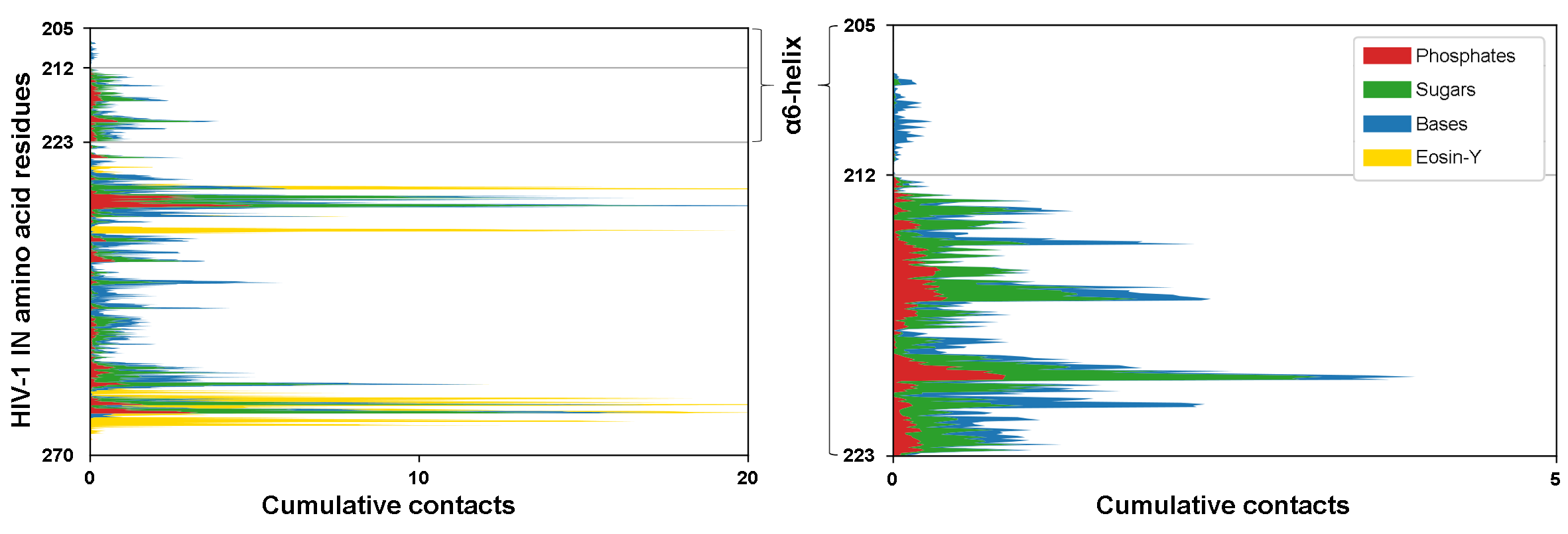

Supplement: Supplementary file 1 [file biomolecules-10-01236-s001.zip › Fig_s5.tif]

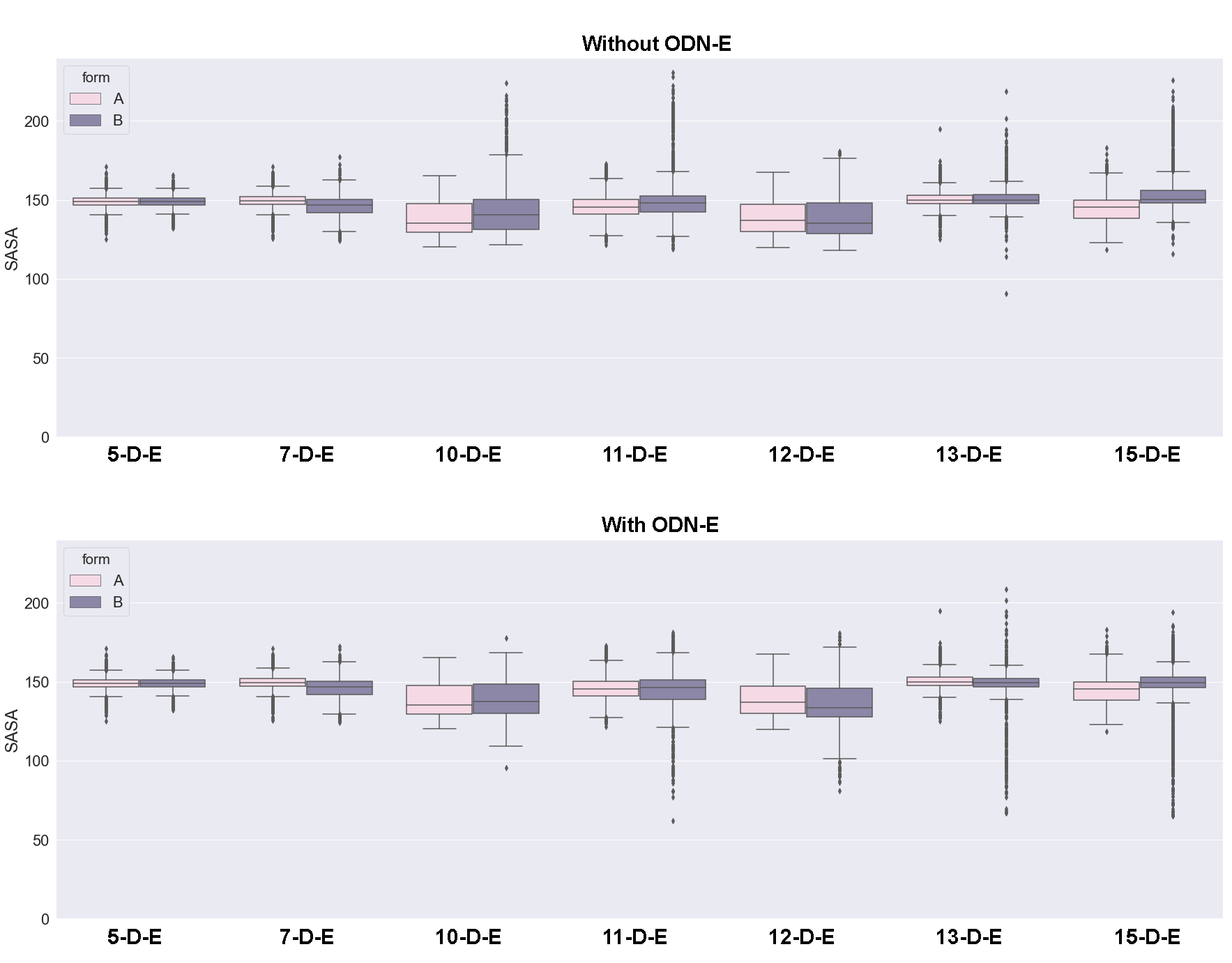

Supplement: Supplementary file 1 [file biomolecules-10-01236-s001.zip › fig_s4.tif]
